# Analysis of chromosomal and genetic disorders in patients with recurrent miscarriages in Saudi Arabia

**DOI:** 10.1186/1471-2164-15-S2-P73

**Published:** 2014-04-02

**Authors:** Rola F Turki, Huda A Banni, Mourad Assidi, Mohammed H Al-Qahtani, Hassan S Abduljabbar, Hassan S Jamel, Abdulrahim A Rouzi, Adel M Abuzenadah

**Affiliations:** 1 Obstetrics and Gynecology Department, King Abdulaziz University, Jeddah, Kingdom of Saudi Arabia; 2 Center of Excellence in Genomic Medicine Research, King Abdulaziz University, Jeddah, Kingdom of Saudi Arabia; 3 KACST Innovation Center for Personalized Medicine, King Abdulaziz University, Jeddah, Kingdom of Saudi Arabia

## Background

Recurrent spontaneous abortion has been reported to occur in 15-20% of all clinically recognizable pregnancies. Numerous studies have reported a clear relationship between the chromosomal abnormalities in parents and recurrent miscarriages and infertility [1-3], however limited data is available from Arabian Peninsula. The main goal of this study was to determine the prevalence of chromosomal abnormalities and correlate them with clinical characteristics of couples with recurrent spontaneous abortions (RSA) in Saudi Arabia.

## Materials and methods

Cytogenetic analysis of 171 consent patients with spontaneous recurrent abortions was performed by the standard method of 72-hour lymphocyte culture and GTG banding. Further validation by conventional PCR and gel electrophoresis was done whenever required.

## Results

Our results showed that 6.43% of patients are carrier of a chromosomal abnormality. The prevalence of mosaicism, balanced translocations, duplications, Robertson translocation, triple X syndrome, and allelic polymorphism were 2.34%, 1.17%, 1.17%, 0.58%, 0.58% and 1.17% respectively. Interestingly, our data showed that women exhibited a higher prevalence to these chromosomal and genetic aberrations than men with female to male ratio of 2.7:1. A significant correlation (*P*<0.05; Table 1) was found between consanguineous marrying families and chromosomal abnormalities in subjects with recurrent abortions, confirming previous findings [4,5]. Surprisingly, 78.6% of young women (≤35 years) with chromosomal aberrations had recurrent miscarriages and therefore infertility problems.

**Table 1:** Clinical features of RSA patients with chromosomal abnormalities in RSA cases

	Normal karyotypes	Chromosomal abnormalities	*P* value*
**Patient gender**			

Male	70	3	0.356

Female	90	8	

**Abortion stage**			

Trimester 1	68	10	0.452

Trimester 2 or 3	19	01	

**Abortion frequency**			

≤3	49	4	0.339

>3	40	7	

**Type of marriage**			

Consanguineous	51	7	**0.046**

Non-consanguineous	109	4	

**Citizenship**			

Saudi	117	6	0.295

Non Saudi	43	5	

*Fisher exact test (*significance value*, *P* <*0.05*)

## Conclusions

The current study reported a strong association between the higher rates of chromosomal abnormalities and recurrent spontaneous abortions. Given the high rate of consanguineous marriages in the Saudi population, these results underline the importance of systematic cytogenetic investigation and genetic counseling preferably at the premarital stage or at least during early pregnancy phase (Preimplantation genetic diagnosis) as recommended elsewhere [6].
